# Functional Analysis of Two *PLA2G2A* Variants Associated with Secretory Phospholipase A2-IIA Levels

**DOI:** 10.1371/journal.pone.0041139

**Published:** 2012-07-17

**Authors:** Holly J. Exeter, Lasse Folkersen, Jutta Palmen, Anders Franco-Cereceda, Jackie A. Cooper, Anastasia Z. Kalea, Ferdinand van’t Hooft, Per Eriksson, Steve E. Humphries, Philippa J. Talmud

**Affiliations:** 1 Centre of Cardiovascular Genetics, Institute of Cardiovascular Sciences, University College London, London, United Kingdom; 2 Atherosclerosis Research Unit, Department of Medicine Solna, Karolinska Institutet, Stockholm, Sweden; 3 Cardiothoracic Surgery Unit, Department of Molecular Medicine and Surgery, Karolinska Institutet, Stockholm, Sweden; University of Tampere, Finland

## Abstract

**Background:**

Secretory phospholipase A2 group IIA (sPLA2-IIA) has been identified as a biomarker of atherosclerosis in observational and animal studies. The protein is encoded by the *PLA2G2A* gene and the aim of this study was to test the functionality of two *PLA2G2A* non-coding SNPs, rs11573156 C>G and rs3767221 T>G where the rare alleles have been previously associated with higher and lower sPLA2-IIA levels respectively.

**Methodology/Principal Findings:**

Luciferase assays, electrophoretic mobility shift assays (EMSA), and RNA expression by RT-PCR were used to examine allelic differences. For rs3767221 the G allele showed ∼55% lower luciferase activity compared to the T allele (T = 62.1 (95% CI 59.1 to 65.1) G = 27.8 (95% CI 25.0 to 30.6), p = 1.22×10^−35^, and stronger EMSA binding of a nuclear protein compared to the T-allele. For rs11573156 C >G there were no luciferase or EMSA allelic differences seen. In lymphocyte cell RNA, from individuals of known rs11573156 genotype, there was no allelic RNA expression difference for exons 5 and 6, but G allele carriers (n = 7) showed a trend to lower exon 1–2 expression compared to CC individuals. To take this further, in the ASAP study (n = 223), an rs11573156 proxy (r^2^ = 0.91) showed ∼25% higher liver expression of *PLA2G2A* (1.67×10^−17^) associated with the G allele. However, considering exon specific expression, the association was greatly reduced for exon 2 (4.5×10^−5^) compared to exons 3–6 (10^−10^ to 10^−20^), suggesting rs11573156 G allele-specific exon 2 skipping.

**Conclusion:**

Both SNPs are functional and provide useful tools for Mendelian Randomisation to determine whether the relationship between sPLA2-IIA and coronary heart disease is causal.

## Introduction

Elevated levels of secretory phospholipases (sPLA2s) show association with several diseases such as coronary heart disease (CHD), rheumatoid arthritis and asthma [1]–[3]. Three sPLA2 enzymes have been identified with links to CHD; sPLA2-IIA (NP_001076000.1), sPLA2-V (GenBank: AAX68682.1) and sPLA2-X (NP_003552.1) [4]. SPLA2 enzymes act by hydrolysing the sn-2 ester bond of phospholipids to release a lyso-phospholipid and a non-esterified free fatty acid (NEFA). Release of the NEFA arachidonic acid (AA) is a key step as a precursor in the production of eicosanoids such as leukotrines, thromboxanes and prostaglandins. It therefore promotes these pro-inflammatory lipid mediators which aid the initiation and maintenance of prolonged inflammatory responses in the body, and are implicated in the development of atherosclerosis [5], [6].

sPLA2 enzymes further contribute to atherogenesis by hydrolysing the outer phospholipid layer of low density lipoprotein (LDL) particles in the circulation, generating small-dense LDL (sd-LDL) particles which can then transverse the endothelial cell layer of the artery wall into the intima, where they are further modified [7]. This modification increases the propensity of the particles to aggregate and bind to proteoglycans, resulting in aggregation of sd-LDL in the intima. The proinflammatory products released by the sPLA2 hydrolysis of phospholipids stimulate monocytes to enter the intima via the endothelial cell wall where they transform into macrophages and take up small dense (sd)-LDL. This leads to foam cell formation and increased atherosclerotic plaque size [7].

The role of sPLA2-IIA in atherogenesis is evident from animal studies. C57BL/6 mice are a natural knockout model for sPLA2-IIA expression. This makes these mice excellent transgenic models for human sPLA2-IIA, as any recorded changes in atherosclerosis attributed to sPLA2-IIA will be due to the human transgene since there is no confounding by endogenous mouse sPLA2-IIA [8]. Mice transgenic for *PLA2G2A* develop atherosclerosis and when macrophages from these mice are transplanted into atherosclerosis-prone knockout mice, either low density receptor *Ldlr−/−* or Apolipoprotein E (*Apoe*) −/−, atherosclerosis is enhanced [9], [10], [11], [12].

The observational studies in prospective cohorts and in the setting of acute coronary syndrome patients support these findings, with both higher sPLA2-IIA levels, and notably higher sPLA2 activity, showing significant association with risk of CAD [9]–[11]. SPLA2-IIA is the most abundantly expressed of the sPLA2 proteins [12], and is mainly expressed in the liver, where it is secreted into the circulation, and in the arterial wall where further modification of sd-LDL takes place.

**Figure 1:**
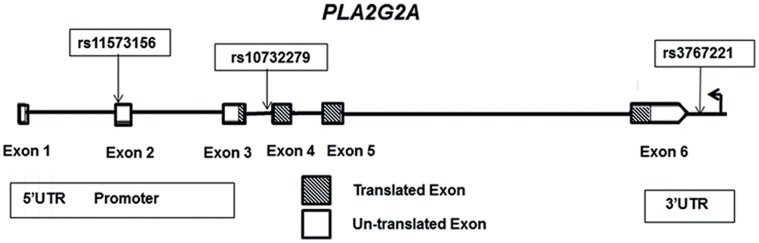
A map of the *PLA2G2A* gene (6 exon transcript). This map identifies rs11573156, rs10732279 and rs3767221 in the 5′UTR, intronic and 3′UTR, respectively. Translated exons are distinguished by hatching. Untranslated exons are unfilled. The gene is normally reverse transcribed.

We previously identified six tagging SNPs (tSNPs), capturing 92% of the variation of *PLA2G2A* (Chr1, NC_000001.10) [13]. These were genotyped in a cohort of patients with type 2 diabetes and their association with sPLA2-IIA levels and lipid traits was examined. Two tSNPs showed strong association with sPLA2-IIA levels. Compared to individuals homozygous for the common C allele for rs11573156 C>G (NC_000001.10:g20306146G>C) in the 5′UTR of *PLA2G2A* (Figure 1), the rare G homozygotes had 2.2 ng/ml higher sPLA2-IIA levels (p = 1.9×10^−14^). For the second variant, rs3767221 T>G (NC_000001.10:g20301781A>C) in the 3′UTR (Figure 1), the opposite effect was seen with the rare G homozygotes having significantly lower (1.8 ng/ml) sPLA2-IIA levels (p = 2.5×10^−10^) compared to the wild-type T carriers [13]. The ultimate aim of our study was to identify a robust functional genetic variant which could be used to determine if the relationship between high sPLA2-IIA levels and coronary heart disease risk was causal or not, a process termed Mendelian Randomization.

## Results

### Allele-specific Expression of *PLA2G2A* in Human Liver

**Figure 2:**
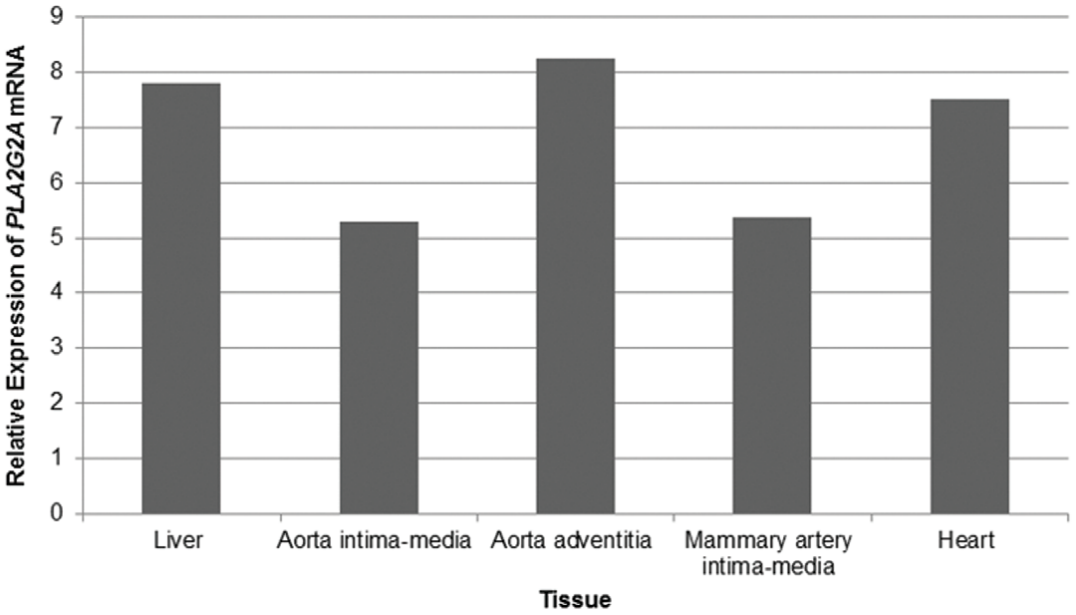
Relative Expression of *PLA2G2A* mRNA in tissues from the ASAP study. This chart shows the relative expression of *PLA2G2A* mRNA across 5 tissues; liver, aorta intima-media, aorta adventitia, mammary artery intima-media and heart, in 223 patients undergoing aortic valve surgery in the Advanced Study of Aortic Pathology (ASAP) study.

**Figure 3:**
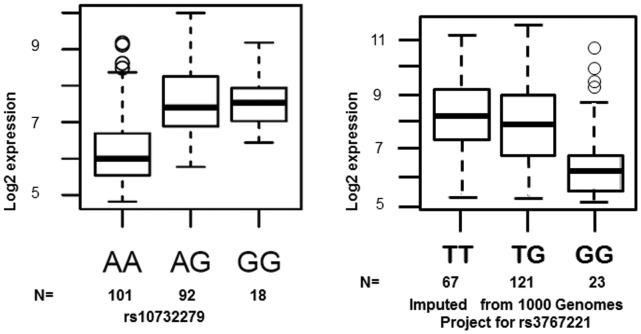
**a. Differential** ***PLA2G2A*** **expression of rs10732279 in the Liver.** Results from the Advanced Study of Aortic Pathology of the overall differential liver expression of *PLA2G2A* according to rs10732279 (A>G) genotype. Rare G carriers have significantly higher *PLA2G2A* expression than common A homozygotes (p = 1.67×10^−17^). **b.** **Predicted Differential** ***PLA2G2A*** **expression of rs3767221 in the Liver.** Results from the Advanced Study of Aortic Pathology relative to the 1000 Genomes Project imputed SNPs for *PLA2G2A*. The figure shows the overall differential expression predicted for rs3767221 (T>G) in the liver. Common T carriers have significantly higher *PLA2G2A* expression than rare G homozygotes (p = 3.6×10^−4^).

To examine the potential allele-specific expression of *PLA2G2A*, we analysed *PLA2G2A* expression data from the ASAP study. Measurements of *PLA2G2A* mRNA expression were investigated in the following tissues; liver, mammary arteries, dilated and non-dilated ascending aorta and heart. *PLA2G2A* mRNA was shown to be most significantly expressed in the aortic adventitia, liver and heart (Figure 2). The most significant allele-specific differential expression of *PLA2G2A* mRNA was found to be in the liver. The SNP rs11573156 was not measured directly on the Illumina Human 610W-Quad Beadarray, so the rs10732279 SNP (Figure 1) was used as a proxy (r^2^ = 0.91 in Caucasian Europeans, HapMap). This proxy SNP showed the greatest overall differential expression of *PLA2G2A* and explained 29.3% of the variance in *PLA2G2A* mRNA in the liver, suggesting that this SNP may have a functional effect. The genotype effect of rs10732279 on *PLA2G2A* liver mRNA is presented in Figure 3a; compared to the common A homozygotes, carriers of the rare G allele had roughly 25% higher expression of *PLA2G2A* (p = 1.67×10^−17^).

Rs3767221 was not covered on the array nor was there a strong proxy for this SNP. However, using the 1000 genomes database we were able to impute rs3767221 and these results predicted an association of rs3767221 with *PLA2G2A*, albeit with less accuracy than for rs11573156. The mach1 r^2^ value was shown to be 0.41. When the differential values of *PLA2G2A* for this imputed SNP were analysed they showed a significant association of p = 3.6×10^−4^ (this was weaker than that seen for rs10732279, the proxy for rs11573156) with carriers of the common allele (T) showing higher expression compared to samples homozygous for the rare allele (G) (Figure 3b).

### Rs3767221 T>G is Associated with Altered *PLA2G2A* Promoter Activity

**Figure 4:**
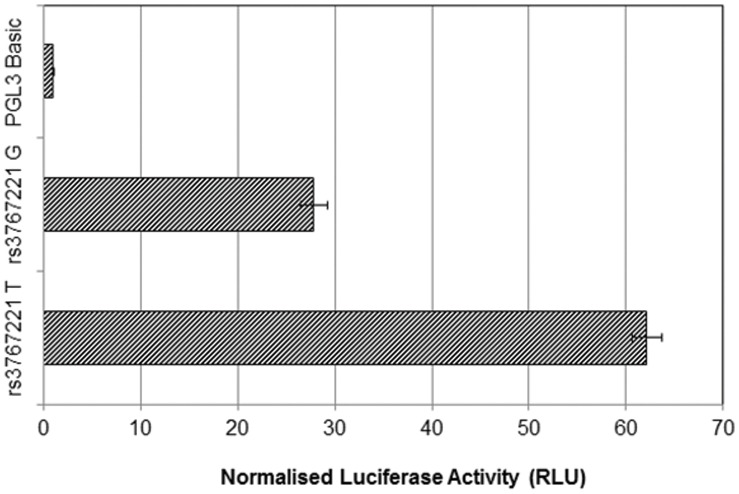
Luciferase Assay Activity Analysis Comparing rs3767221 T >G. The difference in luciferase activity (Relative Light Units) between the T and G alleles of rs3767221 (forward orientation), normalised to pGL3 expression vector. Rs3767221 G (rare) shows a 55% lower Luc activity compared to the T allele (wild type) and is compared to the promoter-free vector pGL3-Basic for baseline reference.

**Figure 5:**
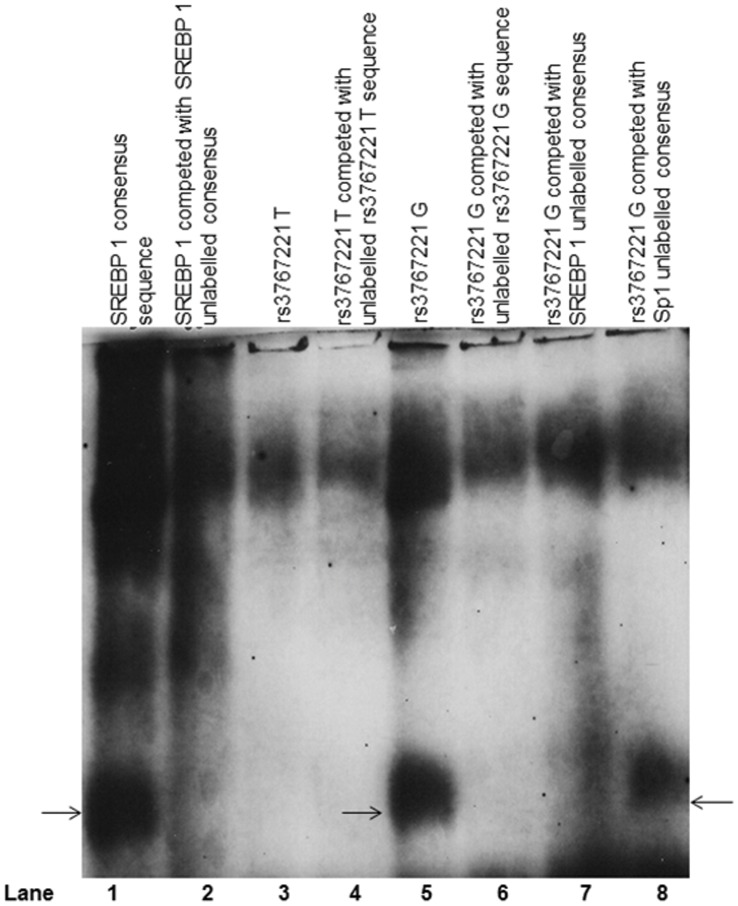
EMSA Analysis of Potential Differential SREBP1 Binding Site for rs3767221 Variants. EMSA image for rs3767221; Lane 1: SREBP 1 specific binding as a positive control. Lane 2 shows the SREBP1 specific bands competed out by unlabelled SREBP1 consensus sequence. Lane 3 and 4 represent rs3767221 T (wild) with (Lane 4) and without (Lane 3) unlabelled competitor with non-specific binding. Lane 5 shows rs3767221 G (rare) appears to have specific binding compared to the wild-type T. Specificity is confirmed in Lane 6 where the band is competed out with an unlabelled rs3767221 G competitor. This confirms differential binding between T and G alleles. Lane 7 shows rs3767221 G competed with unlabelled SREBP 1 and the rs3767221 G specific band is less intense, suggesting SREBP 1 or a related transcription factor may bind at rs3767221 G. Lane 8 shows that the specific band seen with the G allele does not show a loss of intensity with the non-specific competitor Sp1.

Results from the luciferase assays for rs3767221 T>G are shown in Figure 4. A significant difference was seen, with the G allele showing ∼55% lower luciferase activity compared to the T allele (T = 62.1 (95% CI 59.1 to 65.1) G = 27.8 (95% CI 25.0 to 30.6), p = 1.22×10^−35^). An EMSA (Figure 5) confirmed differential binding of nuclear protein between the T and G alleles, illustrated by the additional band seen with the biotin-labelled rs3767221 G, compared to rs3767221 T. This additional band seen with the G variant was competed out by the un-biotinylated G probe, but not the non-specific Sp1 probe, suggesting specific binding of a transcription factor in the presence of the G allele but not the T allele. The difference in transcription factor binding associated with this 3′UTR SNP could influence the rate of transcription and/or mRNA stability, thus affecting *PLA2G2A* expression and levels of sPLA2-IIA.

To investigate this further, rs3767221 G samples were competed with an unlabelled SREBP 1 consensus sequence. For rs3767221 G, a distinct reduction in the intensity of the band was seen, suggesting that SREBP 1 may be involved in the differential binding to the alleles of rs3767221 (Figure 5).

### Rs11573156 C>G is Associated with Alternative Splicing of *PLA2G2A*

**Figure 6:**
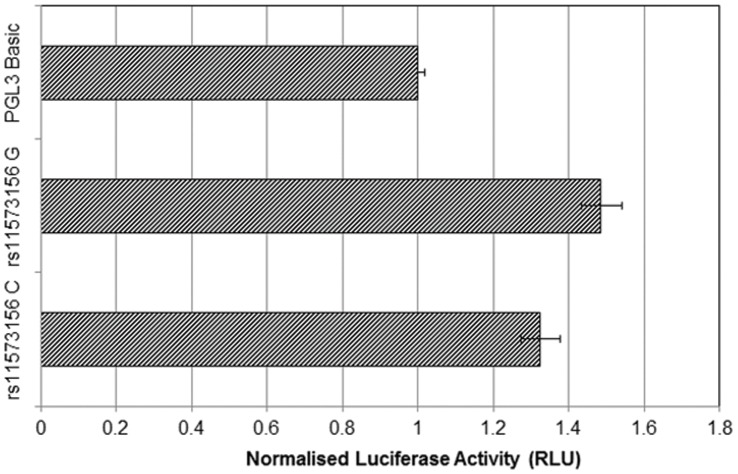
Luciferase Assay Activity Analysis Comparing rs11573156 C >G. The difference in luciferase activity (Relative Light Units) between the C and G alleles of rs11573156 normalised to pGL3-Basic. There is no pre-existing promoter in this vector and both inserted constructs for C and G variants give very low levels of luciferase when compared with pGL3-Basic for baseline reference.

The luciferase assays for rs11573156 C and G alleles showed very low transcription, being in the order of 1.2 to 1.6 relative luciferase units, compared to the promoter-less pGL3-Basic vector alone, which provided a baseline measurement in the order of 1 relative luciferase unit. The G allele shows a modest 10% higher level (C = 1.21 (95% CI 1.13 to 1.30), G = 1.36 (95% CI 1.27 to 1.46), p = 0.02 (Figure 6).

**Figure 7:**
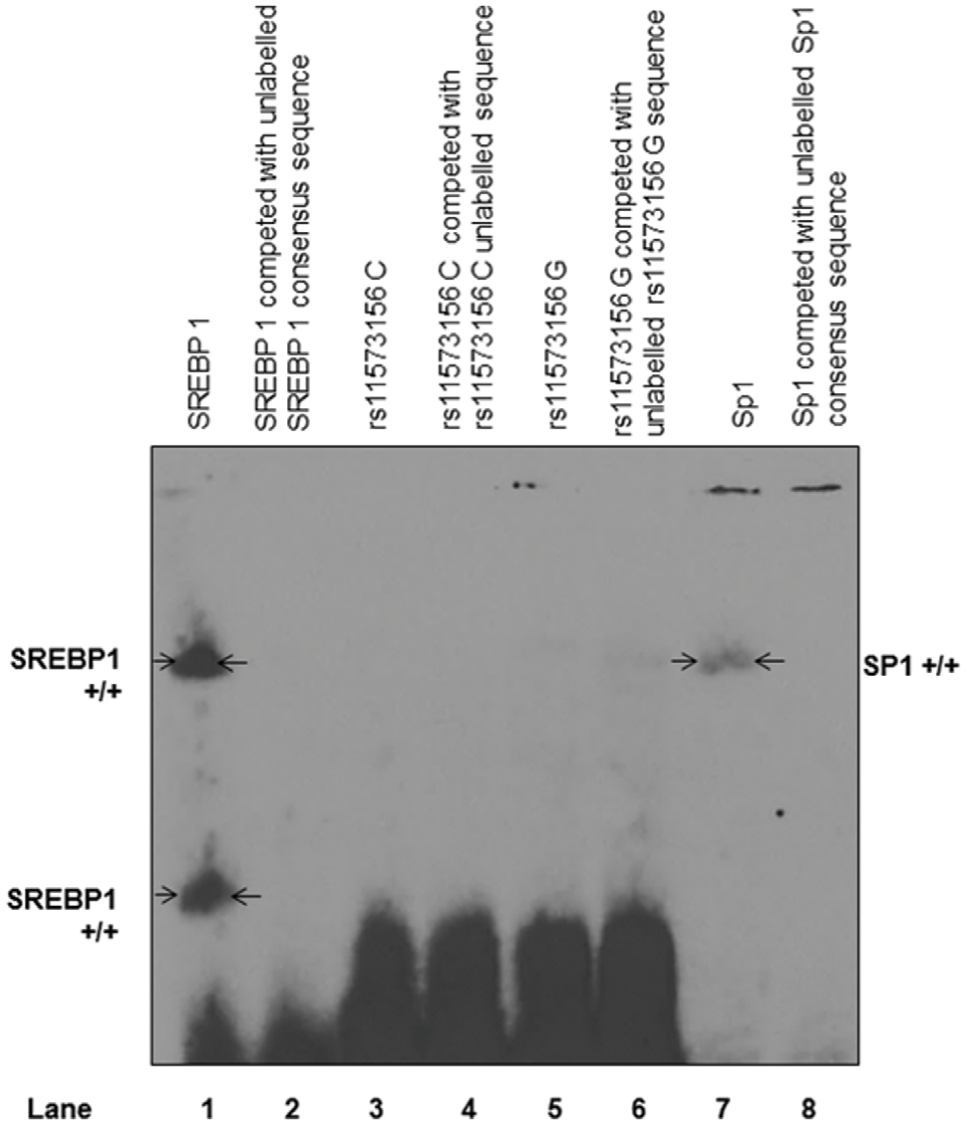
EMSA Analysis of Potential Differential Binding for rs11573156 C and G Variants. EMSA image of rs11573156 C and G alleles showing no transcription factor binding. Lane 1 is SREBP 1 positive control that shows specific bands that are competed out with an unlabelled SREBP 1 consensus sequence in lane 2. Lanes 3 and 4 show rs11573156 C (wild type) labelled and competed with unlabelled C competitor respectively. There is no specific binding. Lanes 5 and 6 represent rs11573156 G (rare) labelled and competed with unlabelled G competitor respectively, again with no specific binding. Lane 7 represents a second positive control with Sp1 specific binding and in Lane 8, labelled Sp1 with unlabelled Sp1 competitor that competes out Sp1 specific bands.

EMSAs for the rs11573156 C or G allele did not show any specific nuclear protein binding (Figure 7). Additionally, the transcription factor binding site prediction algorithm MatInspector did not identify any likely putative differential transcription factor binding sites in the 200 bp region flanking rs11573156. Examination of this region on the UCSC Genome Browser also showed an absence of predicted transcription factor binding sites.

**Figure 8:**
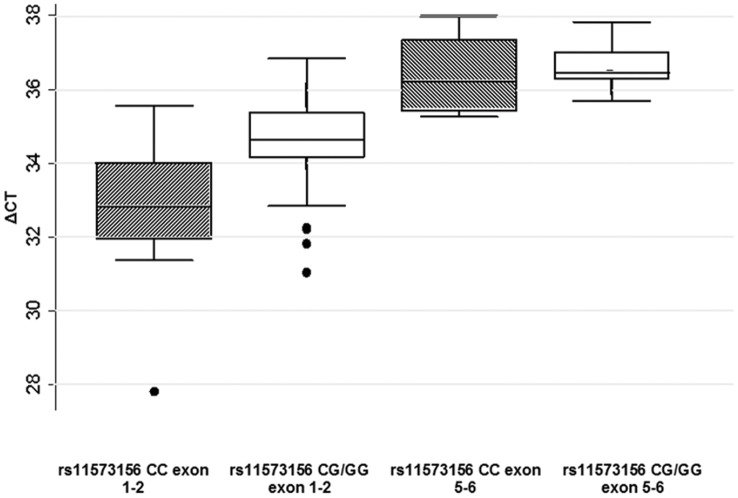
TaqMan Gene Expression Analysis for rs11573156 C and G. TaqMan gene expression assay results for rs11573156 C vs. rs11573156 G expression levels across *PLA2G2A* exon 1–2 and exon 5–6. The X axis scale represents ΔCt, the number of amplification cycles required to reach a pre-determined threshold of fluorescent signal. All samples were normalised to the housekeeping gene *GAPDH*.

Semi-quantitative RT-PCR on lymphocyte cell cDNA samples from individuals of known genotype, across *PLA2G2A* exons 1 and 2 showed a trend towards lower expression levels in the rs11573156 CG and GG individuals, when compared to the 5 rs11573156 C homozygotes, the mean expression in the 7 combined rs11573156 CG and GG individuals showed a non-significant trend towards lower expression of exon 1 and 2 (∼4.9%, p = 0.10) in carriers of the G allele (Figure 8). For exons 5–6, which are present in all six *PLA2G2A* transcripts, no genotype effect on expression levels was seen (p = 0.54; Figure 8). These data were all normalised to a common housekeeping gene, *GAPDH*. These exon-specific gene expression assay results suggest that *PLA2G2A* exon 1 or 2, but not exons 5 and 6, are differentially expressed in a genotype-specific manner according to rs11573156. In order to expand on these results, *PLA2G2A* expression was analysed in the ASAP study.

**Figure 9:**
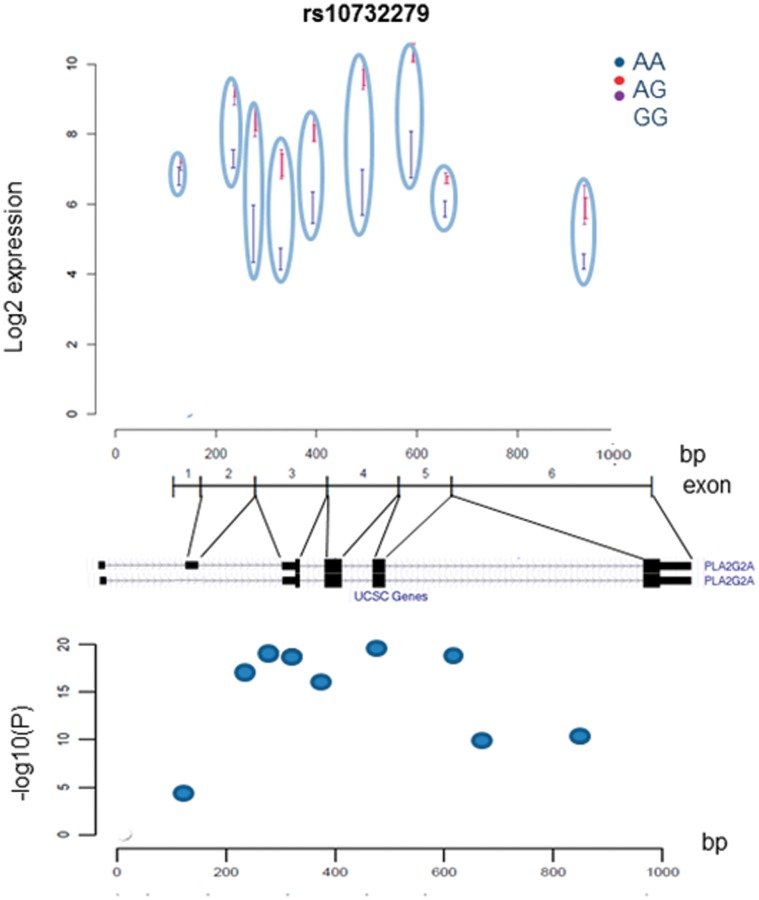
a. ASAP *PLA2G2A* Expression Analysis for Different rs10732279 Genotypes. Results from the ASAP study; liver expression levels of exon-specific *PLA2G2A* probe sets stratified by rs10732279 genotype. *PLA2G2A* is covered by 9 exon-specific probe sets (1 in exon 2, and two each in exon 3–6) **b.** **The Known Protein Coding Transcripts for** ***PLA2G2A.*** Known transcribed *PLA2G2A* transcripts showing alternate exon 2 skipping from the UCSC Genome Browser (UCSC Genome Browser). **c.** ***PLA2G2A*** **Exon-Specific Expression in the Liver.** Results from the ASAP study; Liver expression association between exon-specific *PLA2G2A* probe sets according to rs10732279 genotype. The y-axis give the –log10 (p-value) for the *PLA2G2A* association for rs10732279, using an additive model. These values relate to the exon specific absolute expression levels shown in Figure 9a.

The ASAP study expression array included a single probe set from exon 2, and two probe sets for each of exons 3–6, and Figure 9a shows the rs10732279 allele-specific expression for the nine probes. Rs11573156 is located near the alternately spliced, untranslated exon 2. Of the 6 different *PLA2G2A* transcripts reported, only two are protein coding and only one contains the alternatively spliced exon 2 (Ensemble Genome Browser) (Figure 9b). While rs10732279 genotype did not strongly influence the expression of exon 2, for all exons from exon 3 onwards carriers of rs10732279 G had higher expression than rs10732279 A, consistent with the trend observed in the studies on primary lymphocytes. The p-value for differential expression of exon 2 between the genotype groups was 4.5×10^−5^ while that for the other exons ranged from 10^−10^ to 10^−20^(Figure 9c). Compared with exons 3–6 expression, the low exon 2 expression seen within rs10732279 G carriers could suggest an absence of exon 2 in rs10732279 G carriers.

We examined potential miRNA binding sites in exon 2, based on the hypothesis that if there was a miRNA binding site in this region, in the case of exon 2 being skipped control of gene expression by the miRNA would be affected. According to the analysis from PITA (Segal Lab of Computational Biology), there was one putative miRNA binding site for miR-544 in the region of exon 2, which was in position 96 and had low conservation between species, making it a less likely functional candidate. miRTar predicted 13 miRNAs binding to exon 2, with two of them forming 8 mers and 11 of them 7 mer-m8. As expected due to the use of the same criteria for the algorithms, all the 13 miRTar-predicted miRNAs were also predicted by RegRNA. Furthermore, RegRNA predicted an additional 15 miRNAs relevant to the exon 2 region. Therefore, based on results from these algorithms there is the potential for miRNA binding to affect *PLA2G2A* expression. This requires more detailed examination.

## Discussion

We have previously reported that the *PLA2G2A* 5′UTR SNP rs11573156 and the 3′UTR SNP rs3767221, are independently associated with differences in levels of plasma sPLA2-IIA [13] and here we show that both influence gene expression by acting through different molecular mechanisms.

The difference in luciferase activity between the rs11573156 C and G constructs is modest and of borderline statistical significance, and the lack of mobility shift in the EMSA corroborated our interpretation of the luciferase results that this SNP does not lead to differential binding of a transcription factor. Rs11573156 lies in the region of exon 2. A clue that the genotype effect might be acting through another mechanism came from the observation that exon 2 is not always present in the reported transcripts of the gene (NCBI Reference Sequences). When *PLA2G2A* expression was tested in the limited samples of known genotype available to us, we observed that, compared to expression of exons 5 and 6, which showed no difference in expression by genotype, there was a non-significant lower expression of exons 1 and 2 in G carriers compared to C homozygotes. Our interest was to study the role of sPLA2-IIA in CHD and tissues related to the vasculature; the heart and the liver. Those tissues were available in the ASAP study which provided the eQTLs for our analyses. The expression results were confirmed by the results from the ASAP study which showed that the SNP rs10732279, acting as proxy for rs11573156, was associated with significantly lower differential expression of exon 2 compared to the following four exons of the gene, but significantly increased total mRNA expression across all exons. We acknowledge that the small number of samples and the high ΔCt values we report here for the TaqMan gene expression results cannot act as proof of this exon 2 skipping theory alone. However, taken together with the ASAP study, these results implied that, in the presence of the rare G allele, exon 2 was expressed less well, suggesting that it was preferentially skipped.

Considering the very low levels of luciferase activity (there was no significant increase in activity for either the C allele or the G allele compared to the measure of activity from the promoter free pGL3-Basic) in the luciferase experiment, it is likely that a negative regulatory element exists in the region of exon 2 in the 5′UTR. Fan et al, using deletion mapping of the *PLA2G2A* promoter in reporter gene assays, identified a negative element in the region -1969 to -1624, which incorporates exon 2 (Ensembl Genome Browser). When deleted from the full transcript, reporter gene expression increased by ∼400% [14].

Rs10732279, which showed the most significant association with *PLA2G2A* expression in the ASAP study, is intronic and is not in close proximity to any known functional sites (UCSC Genome Browser). However our SNP of interest, rs11573156, for which it acted as a proxy, is in very close proximity (37 bp) to a known exon skipping site (beginning at chr1∶20306884). One explanation for why skipping of this exon leads to higher expression is the possibility that it harbours a miRNA binding site. MiRNAs are short, non-coding RNA sequences that bind to and interact with mRNA targets in order to post-transcriptionally regulate gene expression. MiRNA target seed sites are thought to occur most frequently in the 3′UTR. However, there is also evidence that they can bind 5′UTR sites and regulate them post-transcriptionally [15], [16]. Although there are fewer prediction algorithms available which include 5′UTRs in their analysis, we have used PITA, miRTar and RegRNA to examine the exon 2 region of *PLA2G2A*, including the rs11573156 site. Using the above sites, which provided overlapping data to a certain extent, we were able to identify potential miRNA binding sites. Absence of the active miRNA binding site, in the case of exon skipping, could account for the loss of post-transcriptional repression.

To examine the possibility that rs11573156 is only marking the true functional SNP in linkage disequilibrium, the LD between rs11573156 and all known exon-boundary SNPs was examined using the 1000 Genomes data and the SNAP Pairwise LD program. There was no other genotyped variant at those positions showing LD with rs11573156 above an r^2^ threshold of 0.7 paired.

There is strong evidence to suggest that rs3767221 T>G in the 3′UTR of *PLA2G2A* is also functional. In this instance we have shown that this is likely to be due to differential transcription factor binding between the wild type and rare variant, with a binding site created by the presence of the rare G allele. The migration of a specific band seen with rs3767221 G corresponded to that of the SREBP 1 positive control, used for all EMSA work. Competition of the G allele with SREBP 1 unlabelled consensus sequence resulted in lower intensity of the specific band, which was not seen with the competition of the G allele with the non-specific Sp1 probe. However, we were unable to confirm SREBP 1 binding using a supershift assay. Future work including a supershift assay and EMSAs of nuclear extracts from cells of known genotype could enhance these results. Prediction by MatInspector suggests a potential cAMP binding protein site in the region of rs3767221 G that is not predicted for rs3767221 T and cAMP is known to effect human sterol regulation, including SREBP 1 binding [17] and to play a role in sPLA2-IIA transcription regulation [18]. The UCSC Genome Browser identifies a dense potential transcription factor binding region in the sequence around rs3767221, which included SREBP 1 or sPLA2 interacting transcription factors such as p300 [19], [20] and LXR alpha [21]. Further experiments are needed to verify and identify the transcription factor binding to this site. Both MatInspector and the UCSC Genome Browser report several other known transcription factor binding regions binding to this site in addition to SREBP 1, including; RXRA, CEBPB, HNF4A and p300, and the Aryl hydrocarbon receptor (AHR).

Observational studies show higher sPLA2-IIA levels associated with the T variant and this is concordant with the higher luciferase activity of the T allele and the higher *PLA2G2A* expression associated with T allele carriers predicted by the ASAP study, so we predict that the transcription factor binding to this region in G allele carriers would act as a repressor.

### Conclusions

The major aim of our study was to determine the functionality of these two SNPs, with a view to validate their use as robust genetic instruments to be used in Mendelian Randomization (MR) analysis. The MR approach uses a genetic variant to validate the causal relationship between a biomarker and outcome, in this case sPLA2-IIA and CHD, by examining the relationship of the SNP with both biomarker and outcome and testing the concordance of effects [22]. We therefore conclude that these two functional variants would be suitable, robust instruments to determine whether sPLA2-IIA is causal of CHD using MR.

## Materials and Methods

### Plasmids and Site Directed Mutagenesis

A 2.27 Kb fragment upstream of the *PLA2G2A* translation start site (GRCh37.p5, chr1∶20,305,406 to 20,307,676) (Ensembl Genome Browser), including wild-type rs11573156 C and the predicted promoter sequence, was amplified from a DNA sample of an individual homozygous for the C allele. The primers were as follows:

Forward, 5′ CCCC GCTAGCTGATCTCTGCCTTCATCTTTGTATG.

Reverse, 5′CCCC AAGCTTCTGCTGGGTGGTCTCAACTTC.

The PCR conditions were; 95°C for 1 minute, then 30 cycles of 95°C for 1 minute, 68°C for 1 minute and 72°C for 1 minute, ending with 72°C for 5 minutes. The amplified fragment was ligated into the pGL3-basic vector upstream of the Luciferase (*Luc*) gene, using the HindIII and NheI restriction sites. Site directed mutagenesis (Stratagene) was used to introduce the rs11573156 G allele.

For rs3767221 T>G, a 303 bp fragment downstream of the *PLA2G2A* protein coding sequence (GRCh37.p5, chr1∶20,301,598 to 20,301,873) (Ensembl Genome Browser) was amplified using the primers:

Forward 5′CGCGGATCCCATGCAGGAGGCACCAGTGTTATCTC.

Reverse 5′ACGCGTCGACGCATGAGAACAAACAGGGGTAGGGG from an individual homozygous for the wild-type allele (T). In this instance the rare G variant construct was created by amplifying a DNA sample from individuals homozygous for the rare variant (G). The PCR conditions were; 98°C for 2 minutes, then 30 cycles of 98°C for 15 seconds, 62°C for 15 seconds and 72°C for 1 minute, ending with 72°C for 5 minutes. PCR fragments were ligated into a pGL3-Promoter expression vector (Promega UK), downstream of the *Luc* gene, using the BamHI and SalI restriction sites. Constructs were transformed into DH5α competent *E.coli*. All products were sequenced by Source BioScience (London, UK) for sequence confirmation.

### Dual Reporter Luciferase Assay

Luciferase activity was measured using the Dual Luciferase Reporter Assay system (TM040, Promega, UK). Huh7 cells (Health Protection Agency Culture Collections) (4×10^4^ cells/well in a 96 well plate format) were transiently transfected with 200 ng of the appropriate plasmid or control vector, 1 ng of pRL-TK co-transfector (Promega, UK), and 0.02 mg Lipofectamine 2000 transfection reagent (Invitrogen) in serum free Opti-Mem serum (Sigma). The cells were lysed 48 h after transfection. Results represent the mean of 3 experiments, each performed in sets of 8 repeats per sample, per plate. All kits and solutions were used as per the manufacturers’ instructions.

### Modified Nuclear Extraction Process

A t-175 flask of Huh7 cells was grown to 100% confluency. The cells were washed with 20 ml of 1×PBS and trypsinised after which, serum-containing medium was added and the cells formed a pellet when centrifuged at 1000 rpm for 5 minutes at 4°C and the supernatant discarded.

The pellet was re-suspended in 5 ml ice-cold buffer A (1 ml 10 M HEPES, pH 7.9; 150 µl 1.5 mM MgCl_2_; 500 µl 10 mM KCl) with added 50 µl of 100× protease inhibitor (Pierce, PERBIO, Protease Inhibitor Cocktail Kit) and incubated on ice for 10 minutes. The cells were then centrifuged for 5 minutes 1000 rpm. The supernatant was discarded. The pellet was re-suspended in 2 ml Buffer A with 20 µl 100× protease inhibitor and vortexed for 30 seconds. The re-suspended pellet mix was centrifuged for 2 minutes at 13,000 rpm. The supernatant was discarded. The pellet was then re-suspended in 400 µl Buffer C (2 ml 20 mM HEPES, pH 7.9; 50 mls 25% v/v glycerol; 10.5 ml 0.42 M NaCl; 150 µl 1.5 mM MgCl_2_; 40 µl 0.2 mM EDTA) and combined with 16 µl of 100× protease inhibitor. This mix was vortexed for 1 minute and incubated on ice for 10 minutes four times. The mix was centrifuged at 13,000 rpm and 4°C for 50 minutes and the supernatant was aliquotted into 50 µl volumes and stored at −80°C.

### Chemiluminescent Electrophoretic Mobility Shift Assay (Chemiluminescent EMSA)

Nuclear extracts were obtained from unstimulated Huh7 cells as described above. Multiple nuclear extracts were tested for the analysis of each SNP. An SREBP 1 probe and SREBP 1 unlabelled competitor were used as controls in each EMSA. An Sp1 unlabelled competitor was used as a non-specific competitor for rs3767221 G EMSAs and labelled and unlabelled Sp1 were used as a secondary control for rs11573156 EMSAs. The consensus sequence for the probes used for SREBP 1 and Sp1 have been detailed previously [23].

The probes used for the EMSAs are detailed below.

For rs11573156 C>G:

Forward wild type 5′-AACCTCCCAGAGGGAGCAGCTATTTAAGGGGAG-3′.

Reverse wild type 5′-CTCCCCTTAAATAGCTGCTCCCTCTGGGAGGTT-3′.

Forward rare variant 5′-AACCTCCCAGAGGGAGGAGCTATTTAAGGGGAG-3′.

Reverse rare variant 5′-CTCCCCTTAAATAGCTCCTCCCTCTGGGAGGTT-3′.

For rs3767221 T>G:

Forward wild type 5′-TGAGCTCAAGCAATCATTGCACTTCAGCCT-3′.

Reverse wild type 5′-AGGCTGAAGTGCAATGATTGCTTGAGCTCA-3′.

Forward rare variant 5′-TGAGCTCAAGCAATCCTTGCACTTCAGCCT -3′.

Reverse rare variant 5′-AGGCTGAAGTGCAAGGATTGCTTGAGCTCA -3′.

For SREBP 1:

SREBP1_F 5′-TTTGAAAATCACCCCATGCAAACTC-3′

SREBP1_R 5′-GAGTTTGCATGGGGTGATTTTCAAA-3′

For Sp1:

Sp1_F 5′-ATTCGATCGGGGCGGGGCGAGC-3′

Sp1_R 5′-GCTCGCCCCGCCCCGATCGAAT-3′

Probes were labelled using the Biotin 3′-End DNA Labelling Kit (Pierce, USA), as described by the manufacturer, and annealed to the complementary labelled oligonucleotide. Each EMSA binding reaction consisted of 2 µl of 10× binding buffer (100 mM Tris, 500 mM KCl; pH 7.5), 1 µg p [dI–dC]), 200 fmol biotin-labelled DNA, and made to a total of 20 µl with dH_2_O, and incubated at 25°C for 30 min. Competition reactions were carried out with 30 min incubation on ice, prior to addition of labelled probes, using 100× unlabelled competitor DNA. Samples were loaded on to a 6% polyacrylamide gel with 5× loading buffer, and electrophoresis was carried out in 0.5× TBE for 5 hours at 120 V at +4°C. The gel was transferred to Hybond nylon membrane (Fermentas) by Southern blot analysis overnight. The membrane was cross-linked using a UV radiation. Protein/DNA complexes are identified with the addition of the biotin label conjugate, horseradish peroxidase using the Chemiluminescent Nucleic Acid Detection Module (Pierce, USA) and visualised on CL-xposure X-ray film.

### TaqMan Gene Expression

The effects of rs11573156 C>G on gene expression was assessed using a TaqMan gene expression assay in a set of 12 healthy Caucasian laboratory volunteers, who had all given informed consent: 5 samples were homozygous for the common allele (CC), there were 4 heterozygous samples (CG) and the frequency of homozygosity of the minor G allele (0.0625) meant it was only possible to identify three GG homozygous samples from our volunteer cohort. The samples were previously genotyped using the TaqMan Genotyping protocol and analysed using the relative quantitation (using comparative Ct) program on the ABI Prism 7900 HT TaqMan machine. Lymphocytes were isolated from 12 samples of 10 ml whole blood (Lymphocyte preparation solution provided by PAA: The Cell Culture Company). RNA was extracted from the isolated cells using the RNeasy Kit (Qiagen). RNA samples were reverse transcribed using Superscript III (Invitrogen). Two TaqMan ABI inventoried gene expression assays, Hs01044022_m1, spanning human *PLA2G2A* exon 1 and 2 and Hs00179898_m1, which spans exon 5 and 6 were used and compared to the housekeeping gene Hs99999905_m1; *GAPDH*, (Applied Biosystems (ABI), California, USA). In all instances the housekeeping gene showed no difference in expression levels across all samples. Each sample was run in triplicate for each assay on the ABI Prism 7900 HT Real Time PCR System. Results were analysed using the REST-384 Version 2 tool.

The RT-PCR conditions were as follows: For 20 µl reaction volumes, 95°C for 2 minutes then, 40 cycles of 95°C for 1 second and 60°C for 20 seconds.

### Advanced Study of Aortic Pathology (ASAP*)* and Evaluation of the Effects of *PLA2G2A* SNPs on *PLA2G2A* mRNA Expression

The Advanced Study of Aortic Pathology (ASAP) recruited 223 patients undergoing aortic valve surgery at the Karolinska University Hospital, Stockholm Sweden [24]. Biobank materials for the ASAP study were generated after informed consent from all participants had been obtained according to the declaration of Helsinki and with approval by the ethics committee of the Karolinska Institute (application numbers 02 to 147 and 2006/784 to 31/1). Tissue biopsies were taken from liver, mammary artery intima-media and dilated and non-dilated ascending aorta intima-media and aorta adventitia and heart during surgery. The medial and adventitial layers of the vascular specimen were isolated by adventicectomy, and incubated with RNAlater (Ambion, Austin, Texas, USA) and homogenised for mRNA extraction as previously detailed [24]. Affymetrix Gene Chip Human Exon 1.0 ST expression arrays were used. MRNA expression was evaluated using RNA pre-processing as previously described [24]. Participants were genotyped using the Illumina Human 610W-Quad Bead array [24], which included 101 SNPs in the region 200 kb up and downstream from the *PLA2G2A* locus. Imputation of SNPs not covered by the Illumina Human 610W-Quad Bead array were imputed for the 1000 genome project. Classification by mach1 suggests >0.3 as the lowest threshold for r^2^. In our set this corresponded to ∼10% mistakes.

### Sequence Analysis of rs11573156 C>G and rs3767221 T>G

#### Prediction of transcription factor binding

Several tools were used to examine the potential functional regions around the two SNPs under investigation. MatInspector is a tool that runs through specified FASTA sequences and was used to identify potential transcription factor (TF) binding sites at and near the two SNP locations. The UCSC Genome Bioinformatics Browser stores information on known genes and their variants as well as identified and suggested transcription factor binding sites, open chromatin regions and known alternate splicing regions. We used these tools, in addition, to check the other identified tSNPs to examine if they might influence functional domains, but these results were negative and we did not continue to investigate them further.

#### Prediction of microRNA (miRNA) binding

In order to predict potential microRNA (miRNA) binding sites in the 5′UTR we input the mRNA sequence for *PLA2G2A*, NM_000300 into three genome-wide target prediction algorithms that focus outside of the 3′UTR. Using the first algorithm, the Probability of Interaction by Target Accessibility (PITA), we searched for possible microRNA targets (among 470 human microRNAs) that form 8 mers with Exon 2. In the miRTar algorithm we searched 1,104 miRNAs across the selected region. The parameters included information such as minimum free energy of hybridization between microRNAs and their predicted target site (MFE of <14 Kcal/mol) and an alignment score of ≥140 based on structural information on the putative hybrid. The third algorithm, RegRNA, identifies miRNA target sites against an input mRNA sequence, which was the RefSeq mRNA ID NM_00300 in our case. As before, we used as cut-off points MFEs <14 Kcal/mol and an alignment score of ≥140 which are estimated from the miRanda algorithm.

### Statistical Analysis

Statistical analysis of luciferase and TaqMan assays was performed using SPSS version 12.0.1 (SPSS, Chicago, IL) and an independent sample T-test was run to determine the p-value, with a threshold of p<0.05. Statistical analysis of microarray expression data was performed using R 2.13.0. P-values are calculated using a linear additive model encoding genotypes numerically. Plotting of location dependent associations in figure 9 was performed using the GeneRegionScan version 1.8.0 [25].
